# Editorial: Modern statistical learning strategies in imaging genetics, volume II

**DOI:** 10.3389/fnins.2023.1337411

**Published:** 2023-12-06

**Authors:** Chao Huang, Rongjie Liu, Bingxin Zhao, Linglong Kong

**Affiliations:** ^1^Department of Statistics, Florida State University, Tallahassee, FL, United States; ^2^Department of Statistics and Data Science, University of Pennsylvania, Philadelphia, PA, United States; ^3^Department of Mathematical and Statistical Sciences, University of Alberta, Edmonton, AB, Canada

**Keywords:** statistical learning, imaging genetic, functional connectivity, transcriptomic, association analysis

As an emerging interdisciplinary study, imaging genetics mainly focuses on using brain imaging techniques to identify and characterize the effects of genetic variations on brain function or structure in order to better understand their impact on behavior and different disease, such as Attention-deficit/hyperactivity disorder (ADHD) (Klein et al., 2017), Autism (Nisar and Haris, 2023), Parkinson's disease (Kim et al., 2017), Alzheimer's disease (Huang et al., 2017), and among others. Although many biomedical studies, such as the Alzheimer's disease neuroimaging initiative (ADNI) study (Mueller et al., 2005), Human Connectome Project (HCP) (Van Essen et al., 2013), and UK BioBank (UKBB) study (Sudlow et al., 2015), are being conducted to collect massive datasets with volumes of imaging [like structural magnetic resonance imaging (MRI), functional MRI, diffusion MRI, and positron emission tomography (PET)], genetic, neurocognitive, and clinical information from increasingly large cohorts, it is still challenging for existing statistical learning approaches to integrate these rich and diverse multi-modal datasets (Huang et al., 2022). These challenges are usually caused by (i) the high-dimensionality of genotype information and the unknown dependencies between them; (ii) the high-dimensionality and irregularity of imaging phenotypes (Liu et al., 2023); and (iii) imaging heterogeneities due to differences in study design, protocol, environment, population, or other hidden confounders (Huang and Zhu, 2022). For example, existing statistical learning methods using regional-wised summary statistics as imaging phenotypes may not account for the spatial configurations of imaging voxels and are sensitive to the choice of region of interest (ROI) atlas, which may cause a loss of prediction accuracy and even lead to inconsistent results. As a follow-up to our first volume on this topic (Huang et al., 2022), this Research Topic includes a group of papers specifically leveraging these massive biomedical datasets to develop new learning approaches in imaging genetics and uncover novel clinical findings.

Integrating transcriptomic data with imaging genetics allows researchers to explore the underlying genetic mechanisms that influence variations seen in medical imaging. It can help in identifying how gene expression patterns relate to differences observed in brain structure, function, or other physiological aspects visualized through imaging techniques. Liu Y. et al. developed an integrative Bayesian scalar-on-image regression model for predicting cognitive outcomes in Alzheimer's disease by integrating voxel-level cortical thickness measurements derived from T1-weighted MRI along with transcriptomics (gene expression) features. Compared to the genetic variants, i.e., single nucleotide polymorphisms (SNPs), the dependencies of high-dimensional and collinear genetic features are well preserved in the transcriptomic information and successfully characterized by a graph Laplacian structure. In addition, the spatial orientation of the voxels in the brain image was captured via a tensor representation for the imaging coefficients, which can uncover patterns and relationships that may be missed by voxel-wise or ROI-based analysis.

The brain activation phenotype in imaging genetics refers to the observable characteristics or patterns of brain activity that are studied in conjunction with genetic variations. It can help in understanding how genetic differences among individuals might influence or correlate with specific patterns of brain activation as observed through various imaging techniques. Jin et al. introduced a Bayesian hierarchical model for detecting influential genetic variants and consistent activation regions using the PET and SNP data from subjects in the ADNI study. They found that their approach can jointly estimate the brain activation regions after accounting for external sources of clinical factors and genetic variation. In particular, it detected important genetic and demographic factors associated with activation intensities inside activation regions. Through applying the proposed method to the AD study, they discovered some important loci correlated to AD-related brain activation regions, which deserved further biological investigations.

Finally, studying resting-state functional connectivity (rs-FC) has become a crucial area of research in the context of psychological diseases or disorders. Liu X. et al. collected resting-state functional MRI data from 31 psychological erectile dysfunction (pED) patients and 31 healthy controls (HCs) and derived the rs-FC to explore the abnormalities of brain function, as well as their relationships with sexual behavior and emotion in pED patients. Altered brain function was found in the medial superior frontal gyrus and caudate-putamen of pED patients, which were associated with sexual function and psychological condition. In addition, Li et al. conducted an image-based meta-analysis to evaluate the effect of different seed selection on rs-FC. They found that the overlap of meta-analytic maps across different seeds' ROIs within the default mode network is relatively low, which means the choice of seed may significantly affect the connectivity results.

Taken together, the studies in this Research Topic include several advanced statistical learning approaches in imaging genetics, and exemplify the potential impact of applying these methods to better understand the roles of brain imaging data and genetic information in mental health and disease.

## Author contributions

CH: Writing—original draft. RL: Writing—original draft. BZ: Writing—review & editing. LK: Writing—review & editing.
